# Supplementary material for: Decorating chromatin for enhanced genome editing using CRISPR-Cas9
Source: Proc Natl Acad Sci U S A. 2022 Dec 2;119(49):e2204259119. doi: 10.1073/pnas.2204259119 (PMC9894255; doi:10.1073/pnas.2204259119)
Supplement: Supplementary file 1 — Appendix 01 (PDF) [file pnas.2204259119.sapp.pdf]

**Supporting Information for**
Decorating chromatin for enhanced genome editing using CRISPR-Cas9

Evelyn Chen[a,b,1], Enrique Lin-Shiao[a,b,1,2], Marena Trinidad[a,b], Mohammad Saffari Doost[a,b], David Colognori[a,b], and Jennifer A. Doudna[a-h,3]

[a]Department of Molecular and Cell Biology, University of California, Berkeley, Berkeley, CA USA 94720
[b]Innovative Genomics Institute, University of California, Berkeley, Berkeley, CA USA 94720
[c]Howard Hughes Medical Institute, University of California, Berkeley, Berkeley, CA USA 94720
[d]Department of Chemistry, University of California, Berkeley, Berkeley, CA USA 94720
[e]Molecular Biophysics and Integrated Bioimaging Division, Lawrence Berkeley National Laboratory, Berkeley, CA USA 94720
[f]California Institute for Quantitative Biosciences, University of California, Berkeley, Berkeley, CA USA 94720
[g]Gladstone Institutes, University of California, San Francisco, CA USA 94158
[h]Gladstone-University of California, San Francisco Institute of Genomic Immunology, San Francisco, CA USA 94158

[1]E.C. and E.L.-S. contributed equally to this work.
[2]Present address: Cystic Fibrosis Foundation, Bethesda MD USA 20814.
[3]Correspondence to: doudna@berkeley.edu.

This PDF file includes:

Figures S1 to S4
Tables S1 to S4
SI References

A)

| | PTM | Biological Process | Presumed Role in DNA Repair |
|---|---|---|---|
| PRDM9 | H3K4me3<br>H3K36me3 | Localization of meiotic recombination hotspots | H3K36me3 and H3K4me3 recruit ZCWPW1 to facilitate repair of SPO11-induced DSBs via HR |
| SETD2 | H3K36me3 | DNA repair by HR, mismatch repair, V(D)J recombination, transcription fidelity | H3K36me3 recruits CtIP to promote DSB resection and RPA and RAD51 foci formation |
| SETMAR | H3K4me3<br>H3K36me2 | DNA repair by NHEJ, supression of chromosomal translocations | H3K36me2 stabilizes NSB1 and Ku70 at DSB sites to promote NHEJ repair. |

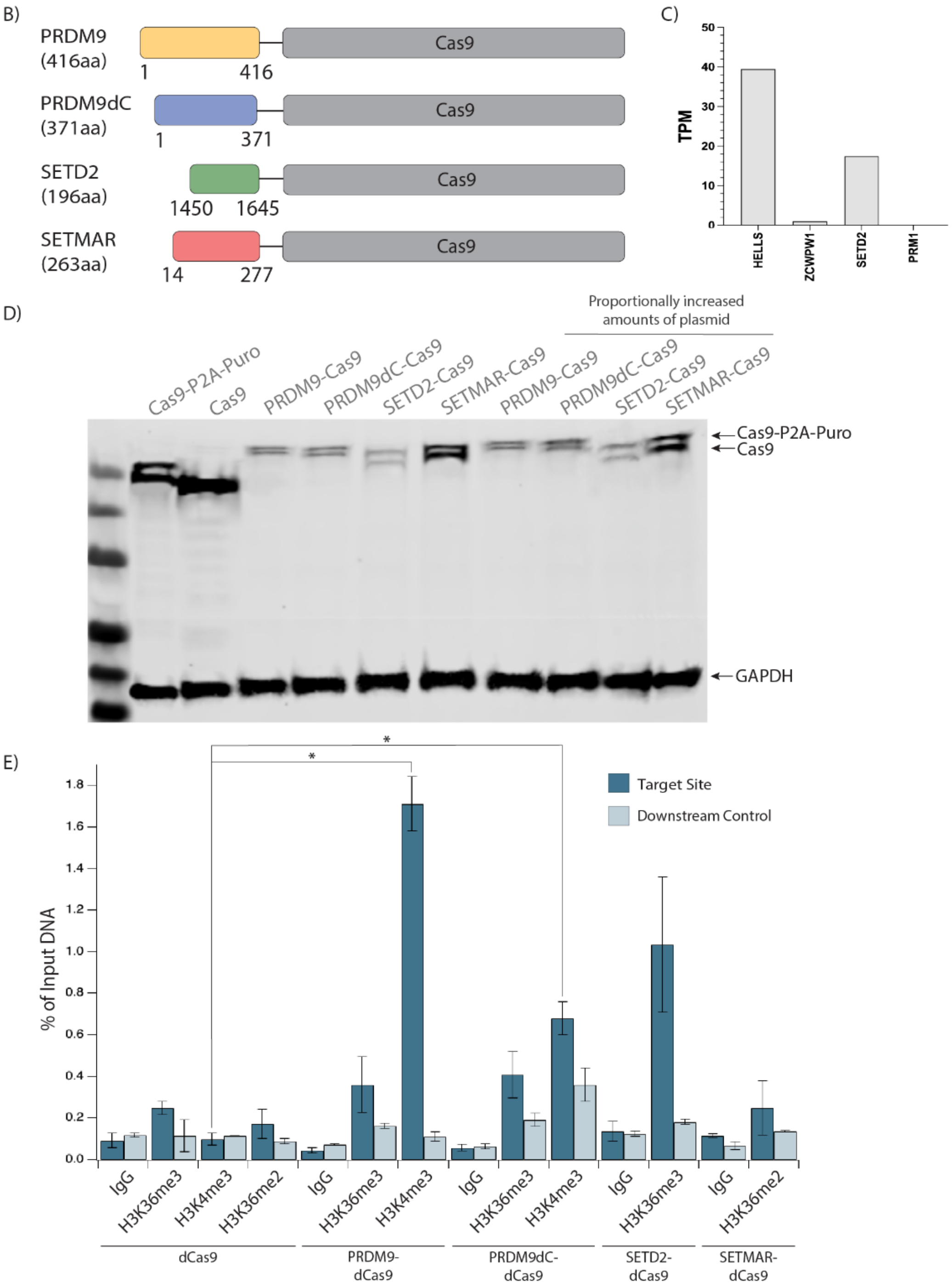

**Fig. S1.** Cas9-methyltransferase fusion protein design to modulate the choice of DNA repair pathway

A) Summary of histone methyltransferases used for Cas9 fusion protein engineering, histone posttranslational modifications (PTM) deposited, biological processes, and their presumed role in DNA repair.

B) Schematic of four Cas9-methyltransferase fusion proteins designed to decorate chromatin to modulate the choice of DNA repair pathway following Cas9-induced DSBs. The histone methyltransferases are fused to Cas9 at the N-terminus, and the amino acid positions of the fused proteins selected for cloning are indicated.

C) Expression levels of relevant genes (HELLS, ZCWPW1) based on RNA-seq experiments in HEK293T cells. Positive control (SETD2) and negative control (PRM1) genes were included.

D) Western blot analysis of fusion protein expression in HEK293T cells. GAPDH antibody was used as a loading control. Double bands were due to incomplete cleavage at the self-cleaving P2A peptide, leading to an uncleaved byproduct (1).

E) H3K36me3, H3K4me3, and H3K36me2 enrichment shown as a percentage of input DNA measured by ChIP-qPCR at site C9 in HEK293T cells at 3 dpt. Cells were transfected with plasmids expressing the dCas9 fusion proteins and sgRNA. Data represents mean ± s.d. (n = 2). $^*P < 0.05$, determined by student's two-tailed t-test.

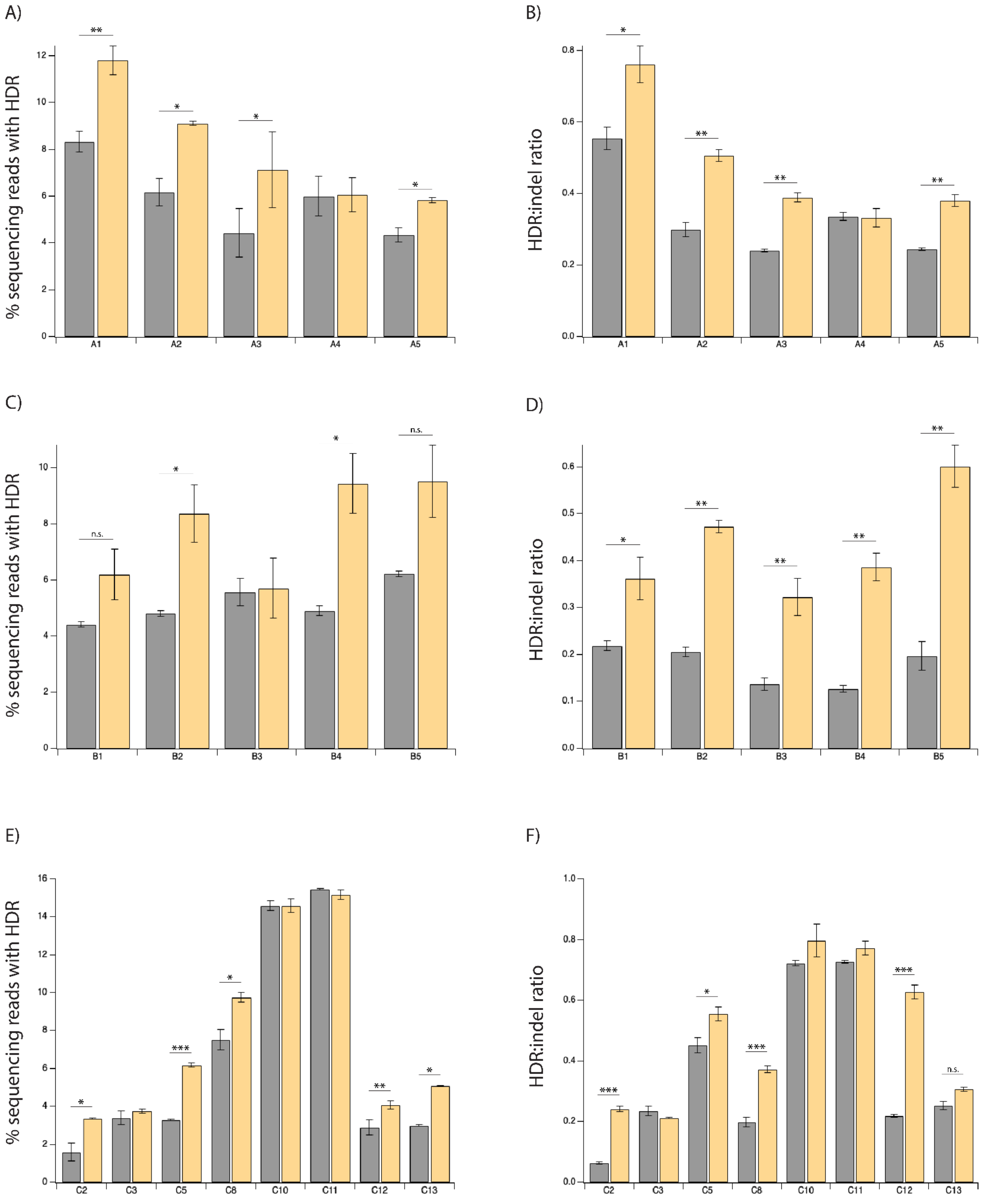
A)
B)
C)
D)
E)
F)
% sequencing reads with HDR
HDR:indel ratio
A1
A2
A3
A4
A5
B1
B2
B3
B4
B5
C2
C3
C5
C8
C10
C11
C12
C13
n.s.
*
**
***

**Fig. S2.** HDR efficiency and HDR:indel ratio improvement by PRDM9-Cas9 at multiple genomic loci

**A)** HDR frequency and **B)** HDR:indel ratio measured by NGS at five genomic loci enriched with H3K36me3 in HEK293T cells transfected with PRDM9-Cas9 and sgRNA with ssODN template.

**C)** HDR frequency and **D)** HDR:indel ratio measured by NGS at five genomic loci enriched with H3K36me3 and H3K4me3 in HEK293T cells transfected with PRDM9-Cas9 and sgRNA with ssODN template.

**E)** HDR frequency and **F)** HDR:indel ratio measured by NGS at eight genomic loci lacking H3K36me3 and H3K4me3 in HEK293T cells transfected with PRDM9-Cas9 and sgRNA with ssODN template.

For A-F, data represents mean ± s.d. (n = 3). *$P < 0.05$, **$P < 0.01$, ***$P < 0.001$, determined by student's two-tailed t-test.

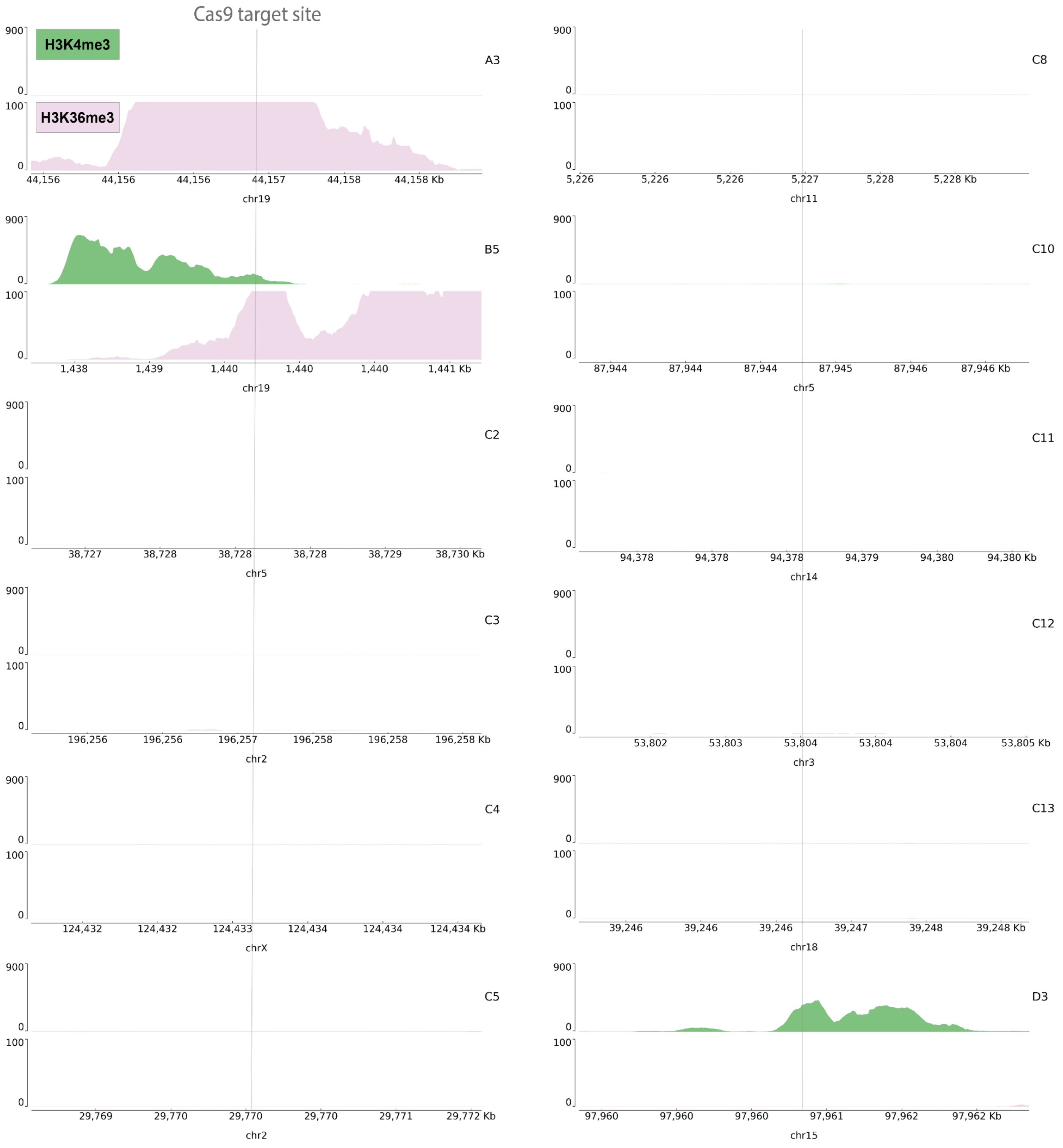


**Fig. S3.** UCSC genome browser tracks showing endogenous levels of H3K4me3 and H3K36me3 enrichment at additional genomic sites

Tracks represent target site ± 1.5 kilobases (kb) in HEK293 cells based on ENCODE ChIP-seq datasets for H3K4me3 (green) and H3K36me3 (pink). Each plot spans 3 kb and the y-axis reports the negative-log p-value for peak enrichment.

A)

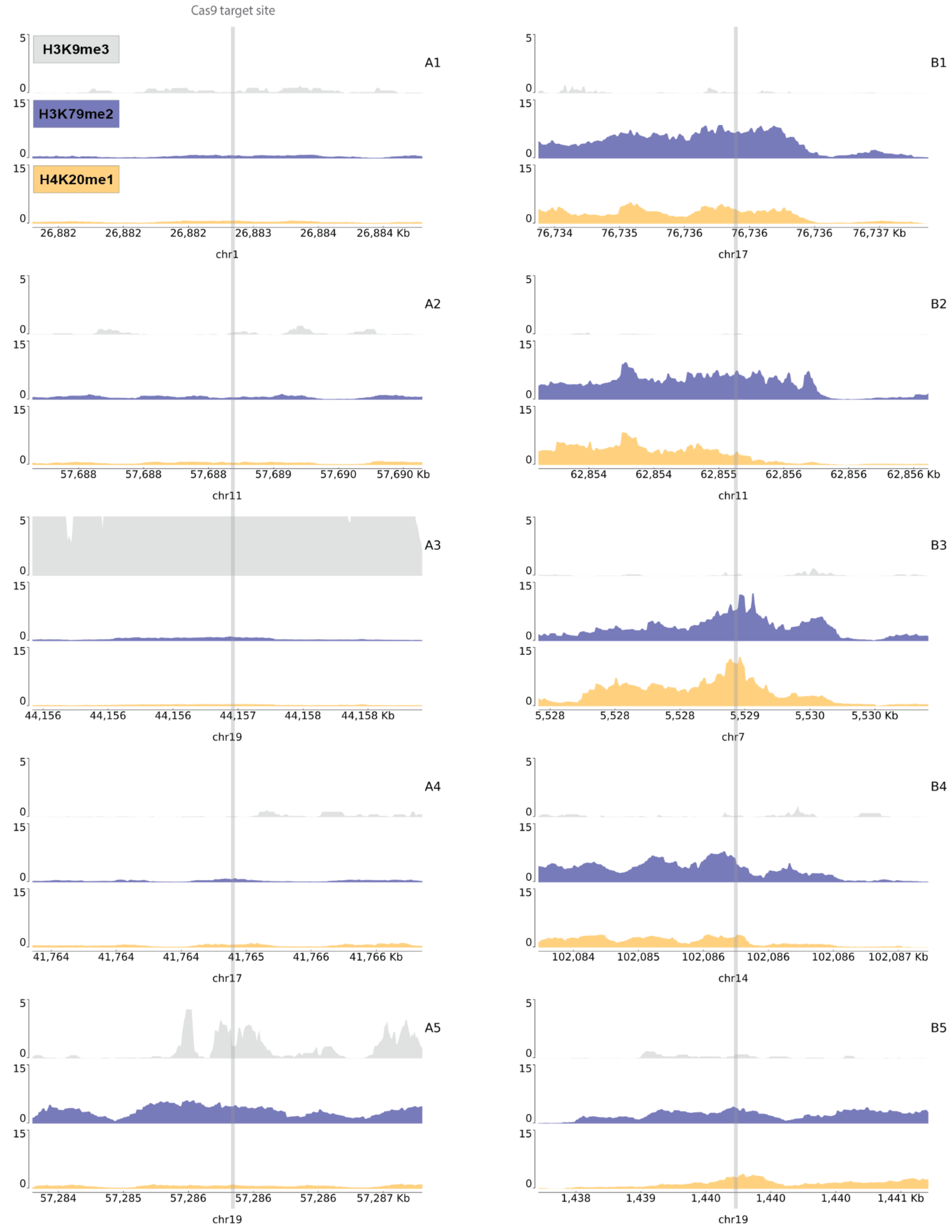
Cas9 target site
H3K9me3
H3K79me2
H4K20me1
A1
B1
26,882 26,882 26,882 26,883 26,884 26,884 Kb
chr1
76,734 76,735 76,736 76,736 76,736 76,737 Kb
chr17
A2
B2
57,688 57,688 57,688 57,689 57,690 57,690 Kb
chr11
62,854 62,854 62,855 62,856 62,856 62,856 Kb
chr11
A3
B3
44,156 44,156 44,156 44,157 44,158 44,158 Kb
chr19
5,528 5,528 5,528 5,529 5,530 5,530 Kb
chr7
A4
B4
41,764 41,764 41,764 41,765 41,766 41,766 Kb
chr17
102,084 102,085 102,086 102,086 102,086 102,087 Kb
chr14
A5
B5
57,284 57,285 57,286 57,286 57,286 57,287 Kb
chr19
1,438 1,439 1,440 1,440 1,440 1,441 Kb
chr19

B)

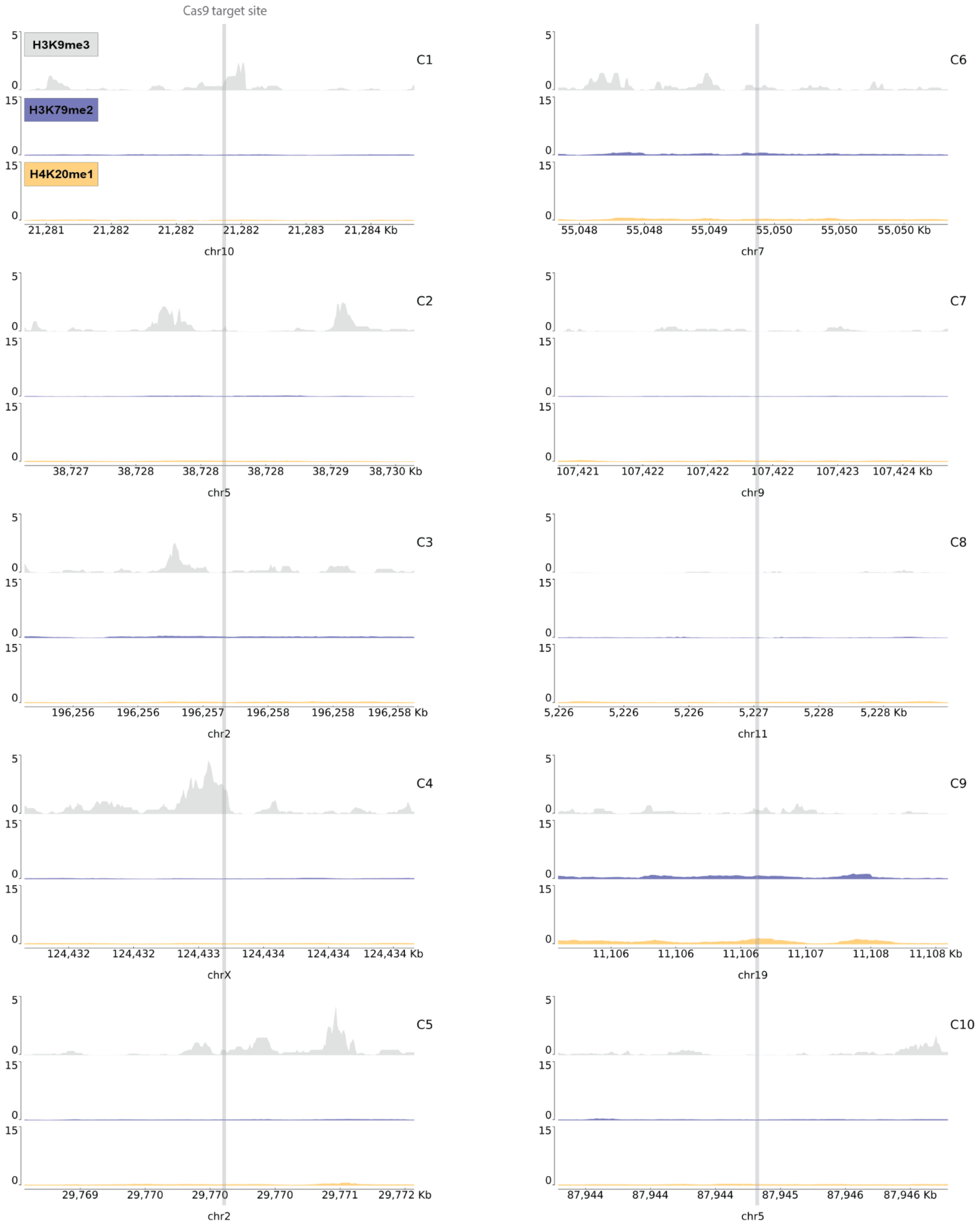

Cas9 target site
H3K9me3
H3K79me2
H4K20me1
C1
chr10
C2
chr5
C3
chr2
C4
chrX
C5
chr2
C6
chr7
C7
chr9
C8
chr11
C9
chr19
C10
chr5

C)

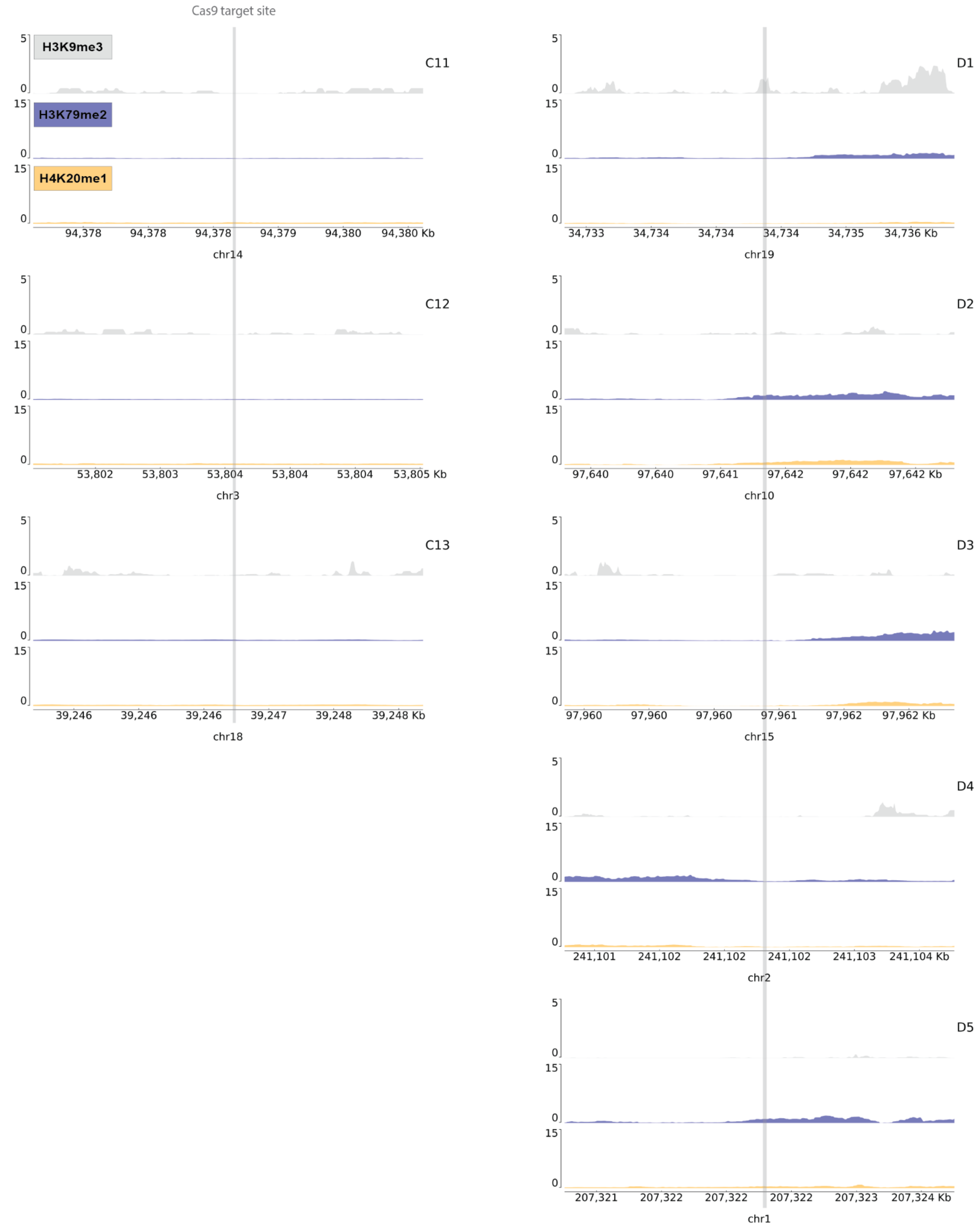

Cas9 target site
H3K9me3
H3K79me2
H4K20me1
C11
94,378
94,378
94,378
94,379
94,380
94,380 Kb
chr14
D1
34,733
34,734
34,734
34,734
34,735
34,736 Kb
chr19
C12
53,802
53,803
53,804
53,804
53,804
53,805 Kb
chr3
D2
97,640
97,640
97,641
97,642
97,642
97,642 Kb
chr10
C13
39,246
39,246
39,246
39,247
39,248
39,248 Kb
chr18
D3
97,960
97,960
97,960
97,961
97,962
97,962 Kb
chr15
D4
241,101
241,102
241,102
241,102
241,103
241,104 Kb
chr2
D5
207,321
207,322
207,322
207,322
207,323
207,324 Kb
chr1

**Fig. S4.** UCSC genome browser tracks showing normalized endogenous levels of H3K9me3, H3K79me2, and H4K20me1 at genomic sites

Tracks represent target site ± 1.5 kilobases (kb) in HEK293 cells based on ENCODE ChIP-seq datasets for H3K9me3 (gray), H3K79me2 (blue), and H4K20me1 (yellow) at **A)** sites A1-A5 and B1-B5, **B)** sites C1-C10, and **C)** sites C11-C13 and D1-D5. Each plot spans 3 kb and the y-axis reports the negative-log p-value for peak enrichment.

**Table S1.** Single guide RNA (sgRNA) sequences and HDR products

| Site | Locus | sgRNA and PAM sequence | Change encoded by ssODN template |
|---|---|---|---|
| A1 | GPN2 | GGACAGCTCTGGATGTCCCAtgg | GGACAGCTCTGGATGTGCCAtgg |
| A2 | ZDHHC5 | GTTGTCACAGACACTGCAGTggg | GTTGTCACAGACACTGGAGTggg |
| A3 | ZNF234 | TATTAAGTGCTGATCTACGAcgg | TATTAAGTGCTGATCTTCGAcgg |
| A4 | JUP | TCAGTCAGCCATGCTCCCCGtgg | TCAGTCAGCCATGCTCGCCGtgg |
| A5 | ZNF460 | GTGGAAGAACTCAATCCTAAggg | GTGGAAGAACTCAATCGTAAggg |
| B1 | SRSF2 | GGAGACCGCAGCTTTAAAGGggg | GGAGACCGCAGCTTTATAGGggg |
| B2 | SNHG1 | CATGGTTTATGCTCTTACAGagg | CATGGTTTATGCTCTTTCAGagg |
| B3 | ACTB | GCTCGTGTGACAAGGCCATGagg | GCTCGTGTGACAAGGCGATGagg |
| B4 | HSP90AA1 | TTCACTGGTTAAGTGAGCGGtgg | TTCACTGGTTAAGTGACCGGtgg |
| B5 | RPS15 | AAAGGCCTCTAAAAACGCAGggg | AAAGGCCTCTAAAAACCCAGggg |
| C1 | NEBL | ACAATGCAAGAGGCTCCGTAggg | ACAATGCAAGAGGCTCGGTAggg |
| C2 | OSMR-DT | ACTGAATTCCTTGAAGACCGggg | ACTGAATTCCTTGAAGTCCGggg |
| C3 | HECW2 | GGATGAGCGTCCCAACCACTggg | GGATGAGCGTCCCAACGACTggg |
| C4 | TENM1 | CTTGGTTGAATCTAATCACGggg | CTTGGTTGAATCTAATGACGggg |
| C5 | ALK | GTCTCTTGCTGGATATGGGAagg | GTCTCTTGCTGGATATCGGAagg |
| C6 | EGFR | GTAATGAAATGAGTTGGGGCagg | GTAATGAAATGAGTTGCGGCagg |
| C7 | | GGCCCAGACTGAGCACGTGAtgg | GGCCCAGACTGAGCAAGTGAtgg |
| | | | GGCCCAGACTGAGCAAGTGATTT (PAM mutation) |
| C8 | HBB | GTAACGGCAGACTTCTCCTCagg | GTAACGGCAGACTTCTCCACagg |
| C9 | LDLR | CAGAGCACTGGAATTCGTCAggg | CAGAGCACTGGAATTAGTCAggg |
| | | | CAGAGCACTGGAATTAGTCAGTG (PAM mutation) |
| C10 | | GAACACAAAGCATAGACTGCggg | GAACACAAAGCAGAGACTGCggg |
| | | | GAACACAAAGCAGAGACTGCGAC (PAM mutation) |
| C11 | SERPINA1 | TGCTGACCATCGACGAGAAAggg | TGCTGACCATCGACAAGAAAggg |
| C12 | CACNA1D | GGAGCAGGAGTATTTCAGTAGTGagg | GGAGCAGGAGTATTTCAGTTGTGagg |
| C13 | | AAGATGCAAGGTTTGTGTCTagg | AAGATGCAAGGTTTGTCTCTagg |
| D1 | ZNF181 | TTGCCCGCTCACCAGCGACGcgg | TTGCCCGCTCACCAGCCACGcgg |
| D2 | PI4K2A | GCATCCCTAAATCCTGCCAGggg | GCATCCCTAAATCCTGGCAGggg |
| D3 | ARRDC4 | GGCAGGAAAGAGTCGCCCGGcgg | GGCAGGAAAGAGTCGCGCGGcgg |
| D4 | MTERF4 | GGCCGAACGCAGCCATAGCGcgg | GGCCGAACGCAGCCATTGCGcgg |
| D5 | CD55 | GCAGCATCGTGTGCTCCACAcgg | GCAGCATCGTGTGCTCGACAcgg |
| BFP-GFP | | GCTGAAGCACTGCACGCCATggg | GCTGAAGCACTGCACGCCGTACG |

**Table S2.** Single-stranded oligonucleotide (ssODN) donor templates

| Site | Template Sequence |
|---|---|
| A1 | CACTAACCAAGCTCCAAAAGTCTGGCTGCCTCTTGGACAGCTCTGGATGTGCCATGGACCCCTGAACACAGCACATCTGCCAGCTCTTGTCCCTTTCATCC |
| A2 | GTCTTACCCCACCCAGGGTGCTTACCTCCACACAGTTGTCACAGACACTGGAGTGGGAACATCGAGGGGGACGGTAAAAGCGGCAGGTGGCACACCATTTC |
| A3 | TATGGTTTCTCTCCTGTGTGGACCATGCAATGATTATTAAGTGCTGATCTTCGACGGAAGTTCTTACCACATGTATCACATTTGAATGGTTTCTCCCCAGT |
| A4 | CGGCTGGGGTGGGAGCTCTGCCCAGCTGCCAGGCTCAGTCAGCCATGCTCGCCGTGGCTGATGGCACCCCTATCCCTGCCCCAGGTGGGATGCAGGCCCTG |
| A5 | AAAACAAGGAGGCCATAAAGAATGGGAGACATGAGTGGAAGAACTCAATCGTAAGGGAACAGGGCAGCAGAGTGAGGGGTAGACGTGGTGACTCCACGCTT |
| B1 | TCTGCAATACTGGCCAATATTCTTTTATCAAACAGGAGACCGCAGCTTTATAGGGGGAAAATGCAGACGTTGGATAAAAACAGCAAGAAATAGTCATTTTC |
| B2 | AGCCAGCGTTACAGTAATGTTCCAGGTAGGTGTACATGGTTTATGCTCTTTCAGAGGAGACCTTGTAGATAACCACTCCATGATGAACACAAAATGACAAG |
| B3 | TAAGACAGTGTTGTGGGTGTAGGTACTAACACTGGCTCGTGTGACAAGGCGATGAGGCTGGTGTAAAGCGGCCTTGGAGTGTGTATTAAGTAGGTGCACAG |
| B4 | ACAGTGAGGACAGACACAGGTAGGCACCTGAACATTCACTGGTTAAGTGACCGGTGGAAGGGTGGGGTGCTGCAACCCTTGGACCCCTGGGGATGCGGTGA |
| B5 | CTCAGACAAGGTGCTGCAGCGTACAGCTCGGGCCAAAGGCCTCTAAAAACCCAGGGGAAGGCAGGTAGGGCGCAGTGGCTCCCGCCTGTGATCCCAGCACT |
| C1 | GGAATTCCTTCATTACAAATATTTATTAGTACCTACAATGCAAGAGGCTCGGTAGGGAGCACAAACCTAAGGAAAGGTGCAAACAAACAAAACCAAGCTTT |
| C2 | GAGCTTATGGTAGAGTTAGAGAAAAGAATGCACAACTGAATTCCTTGAAGTCCGGGGCCTCGACTTAAAATGTGATTATAGGGAGTGGAGTGGAAGGGGTC |
| C3 | TATGCTGTTCCTTTTAATTCTCTGGAGGTTACAGGGATGAGCGTCCCAACGACTGGGTAGAGCAAAGCATTATTTTACTATGGCTTGCCTACATTTGAGGG |
| C4 | TTACATCACTGTCATGTTAGGGTTTTCTGTTATTCTTGGTTGAATCTAATGACGGGGAAGACAGTGCTCTGTTAGGCTTTGGTCTTGACTGGAGTTACTCA |
| C5 | ATTTCTCCAATTCCAGATAGTAAAGAAGCCTTTGGTCTCTTGCTGGATATCGGAAGGGGATTTGTGGATCCAGCTGCTTCTTGAATGAACTTTCATCAAGG |
| C6 | CTTCAGGAGAAATAATGAAGAAAGAGGCGGTTTGGTAATGAAATGAGTTGCGGCAGGAGATTACGGTCATTTCAAGTTATATTCAAATTCTCTTCTTTTTG |
| C7 (lacks PAM mutation) | GCTTCTCCAGCCCTGGCCTGGGTCAATCCTTGGGGCCCAGACTGAGCAAGTGATGGCAGAGGAAAGGAAGCCCTGCTTCCTCCAGAGGGCGTCGCAGGAC |
| C7 (contains PAM mutation) | GCTTCTCCAGCCCTGGCCTGGGTCAATCCTTGGGGCCCAGACTGAGCAAGTGATTTCAGAGGAAAGGAAGCCCTGCTTCCTCCAGAGGGCGTCGCAGGAC |
| C8 | ACTTCATCCACGTTCACCTTGCCCCACAGGGCAGTAACGGCAGACTTCTCCACAGGAGTCAGATGCACCATGGTGTCTGTTTGAGGTTGCTAGTGAACAC |
| C9 (lacks PAM mutation) | ATCAACACACTCTGTCCTGTTTTCCAGCTGTGGCCACCTGTCGCCCTGACTAATTCCAGTGCTCTGATGGAAACTGCATCCATGGCAGCCGGCAGTGTGA |
| C9 (contains PAM mutation) | ATCAACACACTCTGTCCTGTTTTCCAGCTGTGGCCACCTGTCGCACTGACTAATTCCAGTGCTCTGATGGAAACTGCATCCATGGCAGCCGGCAGTGTGA |

| | |
|---|---|
| C10 (lacks PAM mutation) | TTTTCCAGCCCGCTGGCCCTGTAAAGGAAACTGGAACACAAAGCAGAGACTGCGGGGCGGGCC AGCCTGAATAGCTGCAAACAAGTGCAGAATATCTGAT |
| C10 (contains PAM mutation) | TTTTCCAGCCCGCTGGCCCTGTAAAGGAAACTGGAACACAAAGCAGAGACTGCGACGCGGGCCA GCCTGAATAGCTGCAAACAAGTGCAGAATATCTGAT |
| C11 | CATGGGTATGGCCTCTAAAAACATGGCCCCAGCAGCTTCAGTCCCTTTCTTGTCGATGGTCAGCA CAGCCTTATGCACGGCCTGGAGGGGAGAGAAGCAG |
| C12 | ATGGCTATTTCAGGGACCCCCACTGCTTGGGGGAGCAGGAGTATTTCAGTTGTGAGGAATGCTAC GAGGATGACAGCTCGCCCACCTGGAGCAGGTGAGCT |
| C13 | AGAACTGGAATCACTATATGTTTATGTGAAAAAAATGAACCTAGAGACAAACCTTGCATCTTTCAAA AAATTAACCAAAAATAGATTGTAGACATAAATG |
| D1 | TCTGGGCAGTGTCGTCCCCAGAGGTCGGCGGCCGTTGCCCGCTCACCAGCCACGCGGGGCGC CTGCGGGGACGGTGAGGCCCTGCTGAGGACTCCGGCCAG |
| D2 | TTCCGCAGAGCGTCCCCAGCTCCAGTCCTCAGTGGCATCCCTAAATCCTGGCAGGGGCTGGTAC TGTGAAGTTTCTCCCTTTTCCCTCTGGCTGTGCGAAG |
| D3 | GCGACCCCGGCTCCGGGCCTCTGCCGACCTCAGGGGCAGGAAAGAGTCGCGCGGCGGGATGG GCGGGGAGGCTGGGTGCGCGGCGGCCGTGGGTGCCGAGG |
| D4 | AGAAGCCCGCGCGCCCAGCTCGAGCTTACCTGACGGCCGAACGCAGCCATTGCGCGGAGAAGA TGGCAGCAGTTACGGCGCCGGAAGCAGCGGTCCTCCCC |
| D5 | AATGCCACACCTGAAAGAGATGACATGCAAGTTTGCAGCATCGTGTGCTCGACACGGCTGGACTC TGTCGAGAGTGGGGAACGGTCAGCGGTCTGGCCTCC |
| BFP-GFP | GCCACCTACGGCAAGCTGACCCTGAAGTTCATCTGCACCACCGGCAAGCTGCCCGTGCCCTGG CCCACCCTCGTGACCACCCTGACGTACGGCGTGCAGTGCTTCAGCCGCTACCCCGACCACATGA |

**Table S3.** PCR primers for constructing next-generation sequencing libraries

| Site | Primer |
|---|---|
| A1 (for) | TCCCTACACGACGCTCTTCCGATCTAGGAGGGAGCAGGTTTGTCA |
| A1 (rev) | GTTCAGACGTGTGCTCTTCCGATCTGGACACCTACCCATGACCTTG |
| A2 (for) | TCCCTACACGACGCTCTTCCGATCTGATGATTTCCGAGCTCCCCT |
| A2 (rev) | GTTCAGACGTGTGCTCTTCCGATCTGGATGAAGACAATGAAGGTTAAGCA |
| A3 (for) | TCCCTACACGACGCTCTTCCGATCTAGGGCCTTCATACATGCTTCC |
| A3 (rev) | GTTCAGACGTGTGCTCTTCCGATCTACACTTACCACAGTCCTCACAT |
| A4 (for) | TCCCTACACGACGCTCTTCCGATCTGCTGGTGAGTATGATGGCCTTG |
| A4 (rev) | GTTCAGACGTGTGCTCTTCCGATCTCTGTTGCTGGTCAGGTGCTT |
| A5 (for) | TCCCTACACGACGCTCTTCCGATCTGCTATTTGGAGTTCCCTCCC |
| A5 (rev) | GTTCAGACGTGTGCTCTTCCGATCTAGGAAATGAAGAACACCCCCG |
| B1 (for) | TCCCTACACGACGCTCTTCCGATCTAGCTTAGATAATAATGGCTGTTTCG |
| B1 (rev) | GTTCAGACGTGTGCTCTTCCGATCTAGAAACTGGTCCCTGGAGGA |
| B2 (for) | TCCCTACACGACGCTCTTCCGATCTGATGTTCAGCCCACAAGAGC |
| B2 (rev) | GTTCAGACGTGTGCTCTTCCGATCTACATCACTTGAAAGTTCAGCCA |
| B3 (for) | TCCCTACACGACGCTCTTCCGATCTTCTGGTGTTTGTCTCTCTGACTA |
| B3 (rev) | GTTCAGACGTGTGCTCTTCCGATCTGGACATGCAGAAAGTGCAAAG |
| B4 (for) | TCCCTACACGACGCTCTTCCGATCTAGTAACTGTGATCACCAAACATAAC |
| B4 (rev) | GTTCAGACGTGTGCTCTTCCGATCTGATCGTTGGGCAAACACAAA |
| B5 (for) | TCCCTACACGACGCTCTTCCGATCTTGAACTCCTGGGCTCAA |
| B5 (rev) | GTTCAGACGTGTGCTCTTCCGATCTCGTGTGACTGCAGAGATTC |
| C1 (for) | TCCCTACACGACGCTCTTCCGATCTAATGGATGAGCTCTCTGCGG |
| C1 (rev) | GTTCAGACGTGTGCTCTTCCGATCTACACTATGGTAGCCGTCCCT |
| C2 (for) | TCCCTACACGACGCTCTTCCGATCTGCTGAGAAGGACTTCAAGAGAT |
| C2 (rev) | GTTCAGACGTGTGCTCTTCCGATCTCCATATGTGAGAAATAAACAGTCCTAC |
| C3 (for) | TCCCTACACGACGCTCTTCCGATCTAGTAGTTTAGAAAGGAAACTGGCAA |
| C3 (rev) | GTTCAGACGTGTGCTCTTCCGATCTAAAACCTTGCCTGAAAGGCTCA |
| C4 (for) | TCCCTACACGACGCTCTTCCGATCTAGGTGATTCATCCAGAGGTGTA |
| C4 (rev) | GTTCAGACGTGTGCTCTTCCGATCTAAACCACACATCTAGCCTGG |
| C5 (for) | TCCCTACACGACGCTCTTCCGATCTGGTAGTTTACCCTCCTCCTCTA |
| C5 (rev) | GTTCAGACGTGTGCTCTTCCGATCTAGGCATTGATTGTGCTATCTCTA |
| C6 (for) | TCCCTACACGACGCTCTTCCGATCTGGTATCTGCCCAGAAAGCTCTA |
| C6 (rev) | GTTCAGACGTGTGCTCTTCCGATCTGCTGAGTGCAGTGGGAGAGA |
| C7 (for) | GCTCTTCCGATCTGGAAACGCCCATGCAATTAGTC |
| C7 (rev) | GCTCTTCCGATCTCTTGTCAACCAGTATCCCGGTG |
| C8 (for) | GCTCTTCCGATCTCTTGATACCAACCTGCC |
| C8 (rev) | GCTCTTCCGATCTTAAAAGTCAGGGCAGAGCCA |

| | |
|---|---|
| C9 (for) | GCTCTTCCGATCTGCCCTGCTTCTTTTTCTCTGGT |
| C9 (rev) | GCTCTTCCGATCTACCATTAACGCAGCCAACTTCA |
| C10 (for) | GCTCTTCCGATCTCCAGCCCCATCTGTCAAACT |
| C10 (rev) | GCTCTTCCGATCTGAATGGATTCCTTGGAAACAATG |
| C11 (for) | GCTCTTCCGATCTTTTGTTGAACTTGACCTCGGGG |
| C11 (rev) | GCTCTTCCGATCTTCAGGGAGGGAGAGGATGTG |
| C12 (for) | TCCCTACACGACGCTCTTCCGATCTAGGAGATGAACAGCTCCCAACTA |
| C12 (rev) | GTTCAGACGTGTGCTCTTCCGATCTGCTTTTTAGGGGAAGCCCAAGT |
| C13 (for) | GCTCTTCCGATCTTGACACACACACACATGAAAT |
| C13 (rev) | GCTCTTCCGATCTCCAAACGCAAGATCATGTAGA |
| D1 (for) | TCCCTACACGACGCTCTTCCGATCTGTCCGCCCCGCTGTAG |
| D1 (rev) | GTTCAGACGTGTGCTCTTCCGATCTACCCAGGGCCTGAGCC |
| D2 (for) | TCCCTACACGACGCTCTTCCGATCTGGTGAGTGCGGGGGTG |
| D2 (rev) | GTTCAGACGTGTGCTCTTCCGATCTTCCTCCCCCACCAAGTAGG |
| D3 (for) | TCCCTACACGACGCTCTTCCGATCTCCTTACCCTGCCGCGAG |
| D3 (rev) | GTTCAGACGTGTGCTCTTCCGATCTCTCGTCCTCGAACACCAGAC |
| D4 (for) | TCCCTACACGACGCTCTTCCGATCTGGGCCGGAAGTGAAGGAC |
| D4 (rev) | GTTCAGACGTGTGCTCTTCCGATCTACCTGGCCGTGAAACACTC |
| D5 (for) | TCCCTACACGACGCTCTTCCGATCTAGGGAGGGCTCAAAGAGACT |
| D5 (rev) | GTTCAGACGTGTGCTCTTCCGATCTTTCAAGACACAAGCCCCCTT |

**Table S4.** ChIP-qPCR primers

| Site | Primer |
|---|---|
| C7 (for) | CGCCTGTGATGGGCTAATTG |
| C7 (rev) | GGCCTCTCCAGCCTCATTTG |
| C7 control (for) | CCACATTGGCTCCACCCATC |
| C7 control (rev) | CTTGGTTGTGTCACGTGGTT |
| C9 (for) | GTTGGCTGCGTTAATGGTGA |
| C9 (rev) | TCATTTGCAAGCAGCAAGGC |
| C9 control (for) | AGGCTGAGACAGGAGAATCA |
| C9 control (rev) | GAATGCAATGGCACGATATTGG |

**SI References**

1. J. H. Kim, *et al.*, High cleavage efficiency of a 2A peptide derived from porcine teschovirus-1 in human cell lines, zebrafish and mice. *PLOS ONE* **6**, e18556 (2011).
